# A self-made wire snare used in combination with a long transparent cap to remove an obstructive sigmoid fecalith

**DOI:** 10.1055/a-2721-9157

**Published:** 2025-11-10

**Authors:** Ke Liu, Yulin Cheng, Lizhi Yi, Zhengyu Cheng, Huarong Qiu, Zhaojin Yang, Xianfei Zhong

**Affiliations:** 1 Department of Gastroenterology, The Peopleʼs Hospital of Leshan, Southwest Medical University, Leshan, China; 2 Department of General Surgery, Jingyan County Peopleʼs Hospital, Leshan, China

A 68-year-old female was admitted to our hospital with a 5-day history of abdominal pain and vomiting. Computed tomography revealed a mixed-density mass measuring 3.63 cm × 2.65 cm in the sigmoid colon, containing sieve-like low-density areas, along with proximal colonic dilation and fluid accumulation, which are consistent with the presence of a sigmoid colonic fecalith accompanied by colonic obstruction ( Fig. 1 **a** ). The patient chose to undergo endoscopic intervention. Colonoscopy revealed a large black-brown fecalith situated in the sigmoid colon, resulting in complete luminal occlusion and preventing further advancement of the endoscope ( Fig. 1 **b** ). An initial attempt was made to remove the fecalith using a conventional snare; however, the procedure was unsuccessful due to the relatively large size and hardened consistency of the fecalith. Subsequently, a self-made snare fabricated from a zebra guidewire and integrated with a long transparent cap was utilized ( Video 1 ). The fecalith was successfully fragmented, and all resulting fragments were completely removed ( Fig. 1 **c, d** ). No postoperative bleeding or perforation was observed, and the patientʼs abdominal pain significantly improved. She resumed spontaneous bowel movements and was discharged on the second postoperative day.

**Fig. 1:**
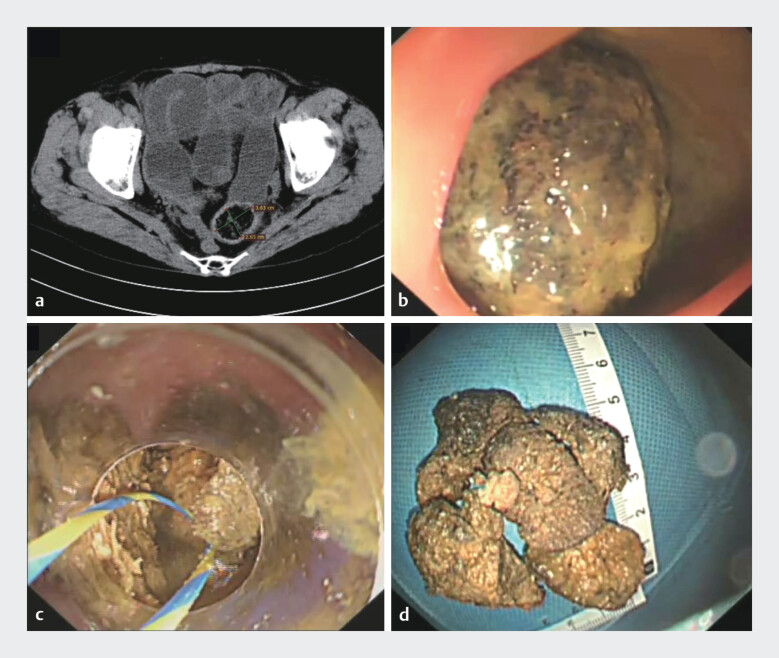
**a** Computed tomography revealed a mixed-density mass measuring 3.63 cm × 2.65 cm in the sigmoid colon. **b** Colonoscopy revealed a large black-brown fecalith situated in the sigmoid colon. **c** A self-made snare fabricated from a zebra guidewire and integrated with a long transparent cap was utilized to remove the fecalith. **d** The fecalith was successfully fragmented, and all resulting fragments were completely removed.

A self-made wire snare used in combination with a long transparent cap to remove an obstructive sigmoid fecalith.Video 1

Fecalith-induced colonic obstruction is not uncommon. However, endoscopic management of fecaliths remains challenging due to their typically large size and hard consistency 1 . Although the combination of a self-made wire snare and a long transparent cap has been reported to be effective in the treatment of giant phytobezoars 2 , its application in the management of colonic fecaliths has not been previously documented. Compared with the stomach, the colon provides a more limited working space, particularly in cases of obstruction where endoscopic visualization is impaired. Our case demonstrates that a self-made snare incorporating a long transparent cap can effectively remove the obstructive sigmoid colonic fecalith.

Endoscopy_UCTN_Code_TTT_1AQ_2AH

## Notes

Endoscopy E-Videos https://eref.thieme.de/e-videos
*E-Videos* is an open access online section of the journal *Endoscopy* , reporting on interesting cases and new techniques in gastroenterological endoscopy. All papers include a high-quality video and are published with a Creative Commons CC-BY license. Endoscopy E-Videos qualify for HINARI discounts and waivers and eligibility is automatically checked during the submission process. We grant 100% waivers to articles whose corresponding authors are based in Group A countries and 50% waivers to those who are based in Group B countries as classified by Research4Life (see: https://www.research4life.org/access/eligibility/ ). This section has its own submission website at https://mc.manuscriptcentral.com/e-videos .
